# Health Equity Assessment Toolkit Plus (HEAT Plus): software for exploring and comparing health inequalities using uploaded datasets

**DOI:** 10.1080/16549716.2018.1440783

**Published:** 2018-07-05

**Authors:** Ahmad Reza Hosseinpoor, Anne Schlotheuber, Devaki Nambiar, Zev Ross

**Affiliations:** a Department of Information, Evidence and Research, World Health Organization, Geneva, Switzerland; b The George Institute for Global Health, Delhi, India; c ZevRoss Spatial Analysis, Ithaca, NY, USA

**Keywords:** Health equity, health inequality, software application, summary measures of inequality, equity monitoring

## Abstract

As a key step in advancing the sustainable development goals, the World Health Organisation (WHO) has placed emphasis on building capacity for measuring and monitoring health inequalities. A number of resources have been developed, including the Health Equity Assessment Toolkit (HEAT), a software application that facilitates the assessment of within-country health inequalities. Following user demand, an Upload Database Edition of HEAT, HEAT Plus, was developed. Launched in July 2017, HEAT Plus allows users to upload their own databases and assess inequalities at the global, national or subnational level for a range of (health) indicators and dimensions of inequality. The software is open-source, operates on Windows and Macintosh platforms and is readily available for download from the WHO website. The flexibility of HEAT Plus makes it a suitable tool for both global and national inequality assessments. Further developments will include interactive graphs, maps and translation into different languages.

## Background

The sustainable development goals (SDGs) have helped retrain the spotlight on one of the most critical issues of our time – reducing inequality [1]. The call to ‘leave no one behind’ is an apt reminder of the centrality and importance of the task before us. A number of SDGs, including Goals 1 (on poverty alleviation), 5 (on gender equality) and 10 (on reducing inequalities within and among countries), directly aim to tackle inequality, while the third goal on health, in placing emphasis on universal health coverage, builds a strong agenda for reducing inequalities in health and ensuring health and wellbeing for all [1,2]. In addition, SDG 17, dealing with strengthening the means of implementation and revitalising global partnerships, makes specific mention of the importance of capacity building to increase the availability of disaggregated data [1], which in turn enables continuous monitoring of health inequalities within countries.

As a key step in advancing these goals, the World Health Organisation (WHO) has placed emphasis on building capacity for measuring and monitoring health inequalities [3]. Capacity building activities have comprised facilitating training workshops in WHO regions and member states as well as developing a number of resources on health inequality monitoring, including the Health Equity Assessment Toolkit (HEAT) [3,4]. HEAT is a software application that facilitates the assessment of within-country health inequalities using the built-in WHO Health Equity Monitor database [4,5]. Last updated in February 2018, HEAT allows users to explore and compare inequalities using disaggregated data and summary measures of inequality, visualised in a variety of customised tables and graphs. The software was developed in tandem with capacity building efforts in countries and regions, and benefited from the feedback of workshop participants and technical experts. The process of developing HEAT is described in detail elsewhere [4].

While HEAT was very well received by countries and programmes, demand emerged for greater customisation of the data used in HEAT. In addition to using the built-in database, users also expressed interest in using their own data, including national- and subnational-level data (e.g. province-, district- and municipality-level data). In response to this demand, the Upload Database Edition of HEAT, HEAT Plus, was developed. Launched in July 2017, HEAT Plus allows users to upload their own databases and assess inequalities at the global, national or subnational level for a range of (health) indicators and dimensions of inequality. The purpose of this article is to introduce HEAT Plus and provide empirical examples of its application in different settings.

## Development

### Software and licensing

Like HEAT [4], HEAT Plus was developed using the free and open source statistical software R (https://www.r-project.org) and the R package shiny (https://cran.r-project.org/package=shiny). It uses R packages that were used in the first version of HEAT (dplyr, ggplot2, grid, gridExtra and RColorBrewer [6–10]) as well as data.table, DT, readxl, and tidyr [11–14]. HEAT Plus source code has been published under the GNU General Public License version 2 or later (https://www.gnu.org/licenses/gpl-2.0) and is freely available through GitHub (https://github.com/WHOequity/HEATPlus-1.0).

### Availability

HEAT Plus is available as a standalone version for download from the WHO website (http://www.who.int/gho/health_equity/assessment_toolkit/en/). Standalone packages can be downloaded as .zip files that also include portable versions of R and Mozilla Firefox, which are required to run HEAT Plus, but do not require any installation. Separate packages are available for Windows and Macintosh operating systems.

### Design and functionality

Once setup, HEAT Plus can be used to assess health inequalities in a variety of different settings and for a range of health indicators and inequality dimensions – depending on the data uploaded. Databases of disaggregated data have to be in a specified format and must be stored as comma-separated values (.csv) or Microsoft Excel (.xls or .xlsx) files in order to be uploaded. The HEAT Plus template illustrates the required content and structure; the user manual provides detailed information on how to prepare disaggregated data according to the HEAT Plus template (both available at http://www.who.int/gho/health_equity/assessment_toolkit/en/).

Table 1 provides an overview of the variables included in the HEAT Plus template, including mandatory, recommended and optional variables. Mandatory variables are essential for the full functionality of HEAT Plus; databases cannot be uploaded if information for these variables is missing for any observation in the dataset. This includes basic information, such as the setting name, as well as additional variables required for the correct calculation of summary measures, including: (1) whether the indicator is favourable or not (variable ‘favourable_indicator’ in the template), (2) the indicator scale (variable ‘indicator_scale’), (3) whether the inequality dimension is ordered or not (‘ordered_dimension’), (4) the subgroup order of the inequality dimension (‘subgroup_order’), and (5) the reference subgroup of the inequality dimension (‘reference_subgroup’). Recommended variables are required for certain calculations or features in HEAT Plus, but databases can be uploaded if information for these variables is missing for some or for all observations in the dataset. For example, users are encouraged to enter the number of people affected or at risk within each population subgroup (variable ‘population’ in the template) as this information is required for the calculation of weighted summary measures. However, if this information is not available to the user, then databases can still be uploaded, but HEAT Plus will only be able to calculate unweighted summary measures. Finally, optional variables may be included to provide additional information, but need not be included in order to upload databases to HEAT Plus. For instance, users may want to enter the 95% confidence intervals (CIs) of subgroup estimates in order to display this information in tables and graphs, although this is not essential for the functionality of HEAT Plus.

**Table 1.** Overview and definition of variables in the HEAT Plus template.

Variable	Definitions and Notes
**Mandatory variables**	
Setting	Setting name (e.g. a country like ‘Indonesia’, or a province like ‘Bali’)
Year	Year (e.g. ‘2016’)
Source	Data source (e.g. ‘DHS’)
Indicator_abbr	Indicator abbreviation (e.g. ‘anc’)
Indicator_name	Indicator name (e.g. ‘Antenatal care coverage’)
Dimension	Dimension of inequality (e.g. ‘Education’)
Subgroup	Population subgroup (e.g. ‘Primary school’)
Estimate	Subgroup estimate
Favourable_indicator	This dummy variable indicates the indicator type. It must be 1 for favourable indicators and 0 for non-favourable (adverse) indicators. *Favourable indicators* measure desirable health events that public health action promotes. They include health intervention indicators, such as antenatal care coverage, and desirable health outcome indicators, such as life expectancy. For these indicators, the ultimate goal is to achieve a maximum level, either in health intervention coverage or health outcome (e.g. complete coverage of antenatal care or the highest possible life expectancy). *Adverse indicators* measure undesirable health events that are to be reduced or eliminated through public health action. They include undesirable health outcome indicators, such as stunting prevalence in children aged less than five years or under-five mortality rate. Here, the ultimate goal is to achieve a minimum level (e.g. theoretically 0 deaths per 1000 live births).
Indicator_scale	This variable indicates the scale of the indicator, such as ‘100’ for indicators reported as percentages or ‘1000’ for indicators reported as rates per 1000 population.
Ordered_dimension	This dummy variable indicates the dimension type. It must be 0 for dimensions with two subgroups (binary dimensions). For dimensions with more than two subgroups, it must be 1 for ordered dimensions and 0 for non-ordered dimensions. *Binary dimensions* compare the situation in two population subgroups (e.g. males and females). *Ordered dimensions* have ordered subgroups that have an inherent positioning and can be ranked. For example, education has an inherent ordering in the sense that those with less education unequivocally have less of something compared to those with more education. *Non-ordered dimensions* have non-ordered subgroups that are not based on criteria that can be logically ranked. Subnational regions are an example of non-ordered groupings.
Subgroup_order	This variable indicates the order of subgroups for ordered dimensions. *For ordered dimensions* (i.e. if ordered_dimension = 1), this variable must be an increasing sequence of integers starting with the value 1 for the most-disadvantaged subgroup. For example, for education with three subgroups, the most-disadvantaged subgroup ‘No education’ will be assigned the value 1, ‘Primary school’ will be assigned the value 2 and the most-advantaged subgroup ‘Secondary school +’ will be assigned the value 3. *For non-ordered dimensions and binary dimensions* (i.e. if ordered_dimension = 0), this variable must be 0.
Reference_subgroup	This variable indicates the reference subgroup for non-ordered dimensions and binary dimensions. *For ordered dimensions* (i.e. if ordered_dimension = 1), this variable must be 0. *For non-ordered dimensions and binary dimensions* (i.e. if ordered_dimension = 0), a reference subgroup can be chosen. A reference subgroup can be chosen by assigning the value 1 to that subgroup and 0 to all other subgroups. For example, for subnational regions (with more than two subgroups), the capital city can be chosen as the reference subgroup. For place of residence (urban vs rural), urban can be chosen as the reference subgroup.
**Recommended variables**	
se	Standard error of subgroup estimate
Population	The number of people affected or at risk within that subgroup (e.g. weighted sample size by subgroup in household surveys).
Setting_average	Setting average
iso3	ISO3 country code for country-level data (e.g. ‘IDN’ for Indonesia).
**Optional variables**	
95ci_lb	95% confidence interval lower bound of subgroup estimate.
95ci_ub	95% confidence interval upper bound of subgroup estimate.
flag	Flag of subgroup estimate, indicating notes or observations relevant to the analysis. For example, if a subgroup estimate is based on a very small number of cases, this could be indicated in the flag.

Provided databases have been prepared in the correct format, HEAT Plus will be able to upload the disaggregated data and calculate up to 15 summary measures of inequality. Table 2 provides an overview of which summary measures can be calculated for which dimension of inequality, depending on the data included in the uploaded database. Disaggregated data and summary measures will be stored in HEAT Plus under a user-specified name. Stored databases can be opened when returning to HEAT Plus.

**Table 2.** Overview of summary measures of inequality in HEAT Plus^a^.

	Dimension of inequality		
Summary measure	Dimension with 2 subgroups	Non-ordered dimension with more than 2 subgroups	Ordered dimension with more than 2 subgroups
**Absolute measures**			
Absolute concentration index (ACI)			✓*
Between-group variance (BGV)		✓*	
Difference (D)	✓	✓	✓
Mean difference from best performing subgroup (MDB)		✓*	
Mean difference from mean (MDM)		✓*	
Population attributable risk (PAR)	✓*	✓*	✓*
Slope index of inequality (SII)			✓**
**Relative measures**			
Index of disparity (IDIS)		✓*	
Index of disparity (weighted) (IDISW)		✓*	
Mean log deviation (MLD)		✓*	
Population attributable fraction (PAF)	✓*	✓*	✓*
Ratio (R)	✓	✓	✓
Relative concentration index (RCI)			✓*
Relative index of inequality (RII)			✓**
Theil index (TI)		✓*	

^a^Please refer to the HEAT Plus technical notes for the further information.

*Note that this summary measure can only be calculated if, in addition to the subgroup estimates, information about the number of people affected or at risk within each subgroup have been entered in the uploaded database (variable ‘population’ in the HEAT Plus template).

**Note that this summary measure can only be calculated if, in addition to the subgroup estimates, standard errors of subgroup estimates and information about the number of people affected or at risk within each subgroup have been entered in the uploaded database (variables ‘se’ and ‘population’ in the HEAT Plus template).

**Figure A1.**
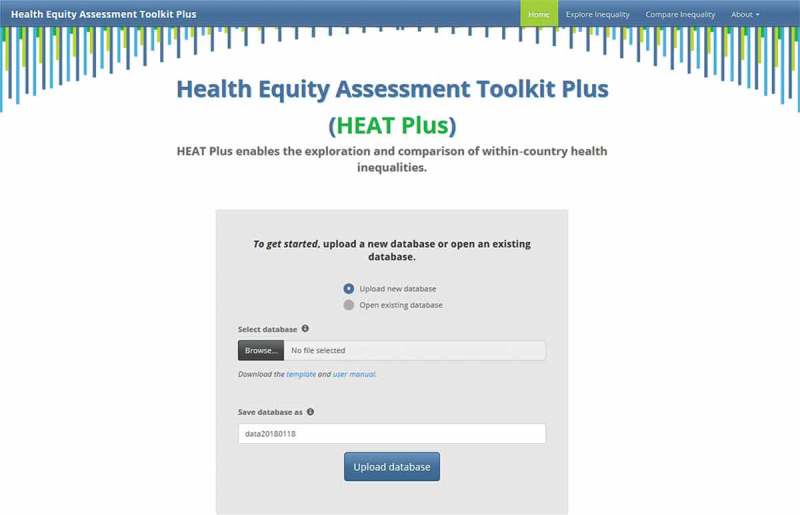
Screenshot of the HEAT Plus homepage.

**Figure A2.**
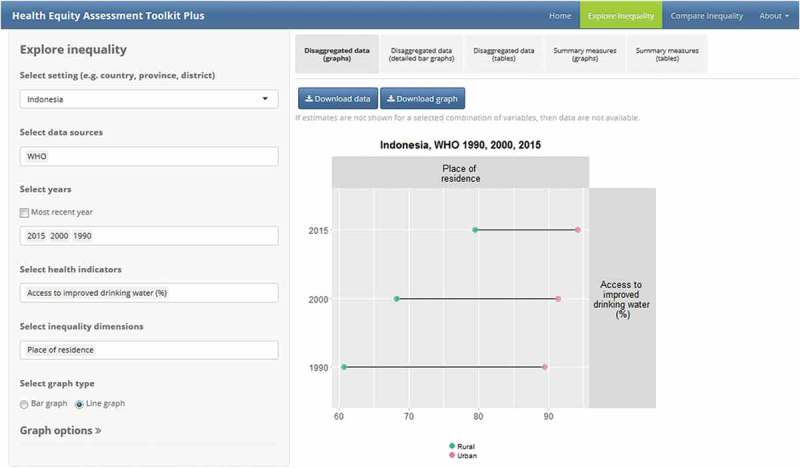
Screenshot of the Explore Inequality tab and subtabs in HEAT Plus.

**Figure A3.**
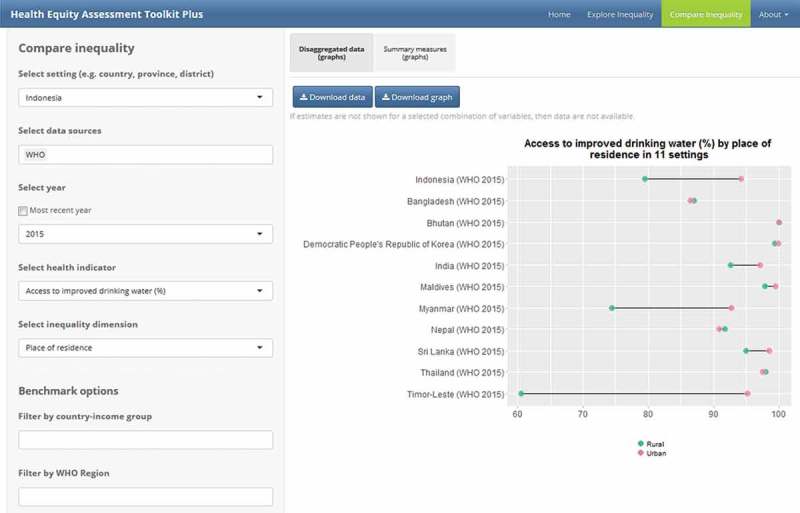
Screenshot of the Compare Inequality tab and subtabs in HEAT Plus.

Like HEAT [4], HEAT Plus is organised around four main tabs: Home, Explore Inequality, Compare Inequality and About. Home is the starting point for any assessment in HEAT Plus, where users can upload new databases or open existing databases (see Figure A1). Under About, users can access further information about HEAT Plus, including the user manual and technical notes as well as software, version, licence and feedback information, and the acknowledgements. The two key tabs, Explore Inequality and Compare Inequality, allow users to undertake health equity assessments using disaggregated data and summary measures  (see Figures A2 and A3). Both tabs comprise a number of subtabs, each displaying the data in a graph or table that can be customised using a selection menu on the left. The subtabs are identical in HEAT and HEAT Plus; details of the subtabs included in HEAT version 1.0 have been described elsewhere [4]. However, a number of improvements have been made since the first release of HEAT, which are highlighted in the following section.

### New features and improvements

First, a new subtab has been added under Explore Inequality, named Disaggregated data (detailed bar graphs). The tab features horizontal bar graphs showing data for population subgroups in a selected setting of interest and is the preferred option for assessing dimensions with a large number of subgroups, such as data by districts. Graph Options in the left panel allow users to modify the graphs: bars can be sorted by subgroup name or subgroup estimate (in ascending or descending order); the median and/or setting average can be shown as vertical lines in the graph; one or more subgroups may be highlighted in the graph; the graph height and width and the axis range can be modified; graph and axis titles can be added. As in HEAT, the graph and the data represented in the graph may be downloaded as .pdf/.png/.jpg and .csv/.tsv files, respectively. Second, graph and table options in all subtabs now make use of accordion-style menus, allowing users to hide or show these options at their convenience. Third, the order of subtabs under Explore Inequality has been changed and graphs are now listed before tables (this was done in response to users’ preference).

## Use

HEAT Plus can be used for monitoring health inequalities at global and national levels. Preparing data for upload to HEAT Plus requires good data management skills and familiarity with Excel. Once prepared and uploaded, data can be used for health inequality assessments in HEAT Plus by anyone with data interpretation competence, such as technical staff (for example, in ministries of health), public health professionals, policy makers, researchers, students and others. The following examples illustrate prior applications of HEAT Plus.

### Global example

**Figure 1.**
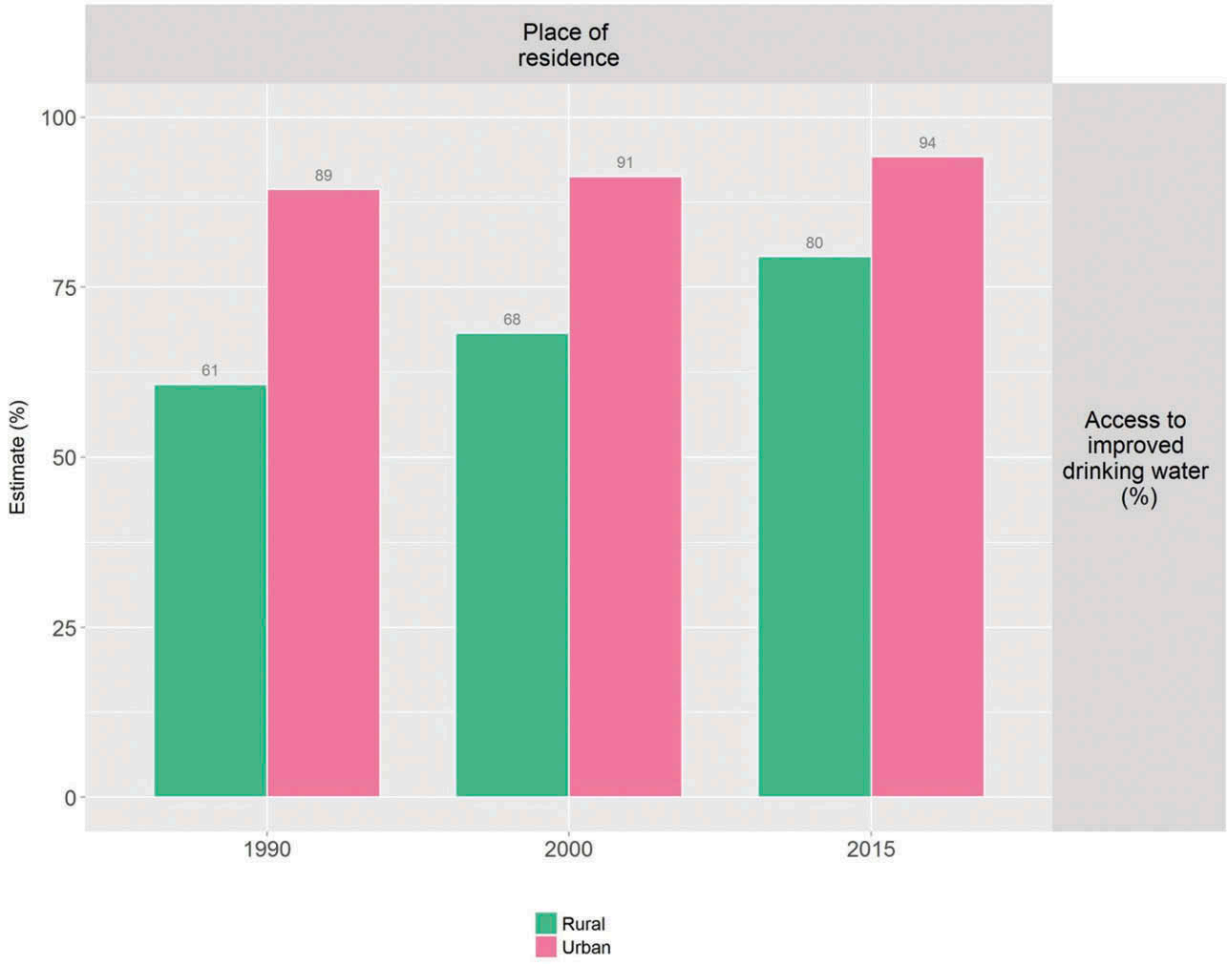
Access to improved drinking water in Indonesia, by place of residence (WHO 1990, 2000, 2015).

**Figure 2.**
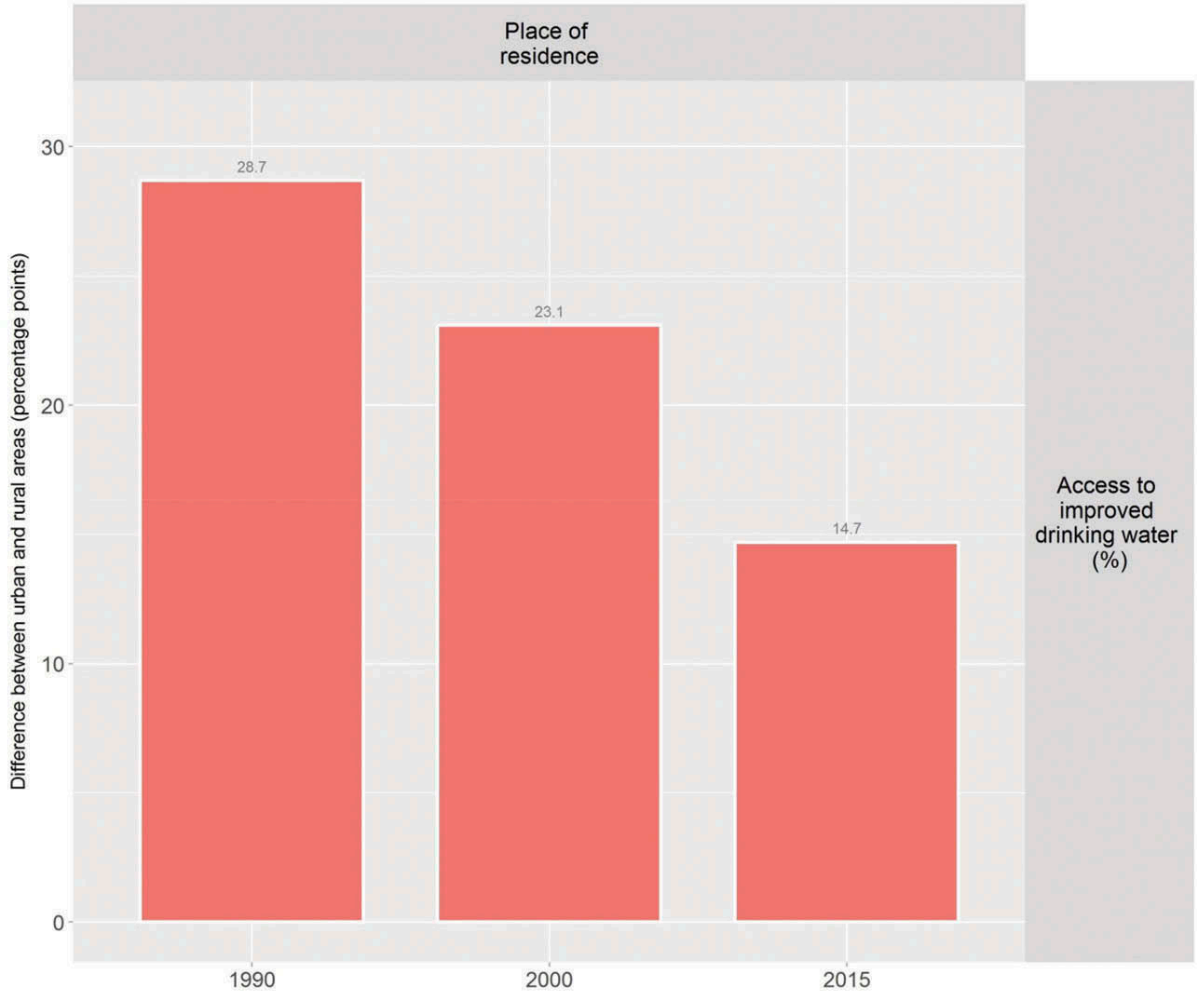
Access to improved drinking water in Indonesia: absolute place-of-residence-related inequality (WHO 1990, 2000, 2015).

**Figure 3.**
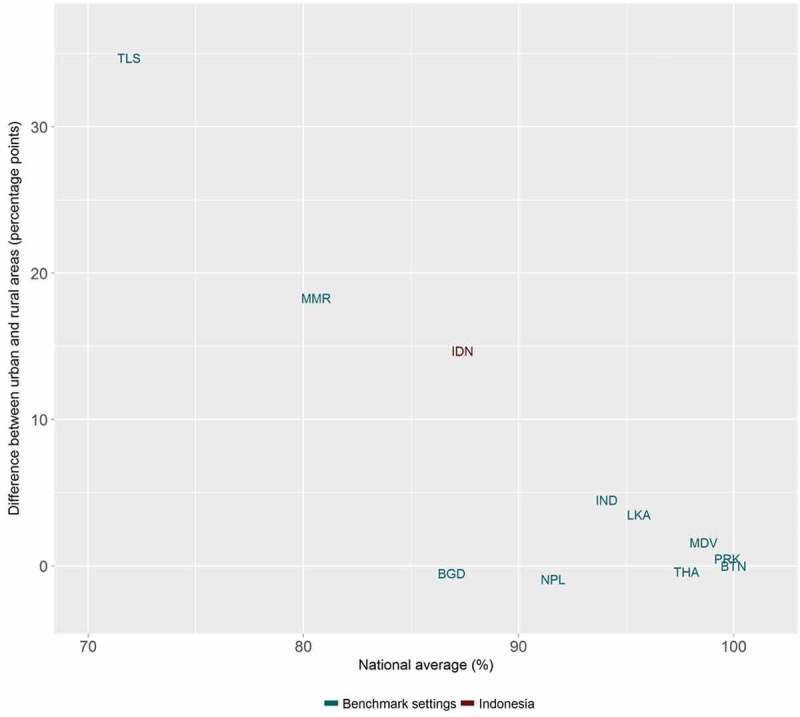
Access to improved drinking water in 11 low- and middle-income countries from the WHO South-East Asia Region: national average and absolute place-of-residence-related inequality (WHO 2015).

At the global level, HEAT Plus has been used to assess the state of inequality in access to improved drinking water in WHO Member States. Data disaggregated by place of residence (urban/rural) for 192 countries were taken from the WHO Global Health Observatory data repository [15], prepared according to the HEAT Plus template and uploaded to HEAT Plus. The Explore Inequality tab was then used to explore the latest situation of inequality and the change in inequality over time within a selected country of interest. The Compare Inequality tab was used to compare the situation in that country with the situation in other countries from the same or different country income groups and/or WHO regions. Looking at Indonesia, for example, analyses in HEAT Plus showed that access in both urban and rural areas increased between 1990 and 2015 and that the absolute difference between the two areas was halved during this period. However, a considerable gap between urban and rural areas remained in 2015 (see Figures 1 and 2). When comparing Indonesia with other low- and middle-income countries from the WHO South-East Asia region, it was found that some countries, such as Bhutan and the Democratic People’s Republic of Korea, achieved almost universal coverage, while other countries reported even larger inequalities than Indonesia (see Figure 3).

### National example

**Figure 4.**
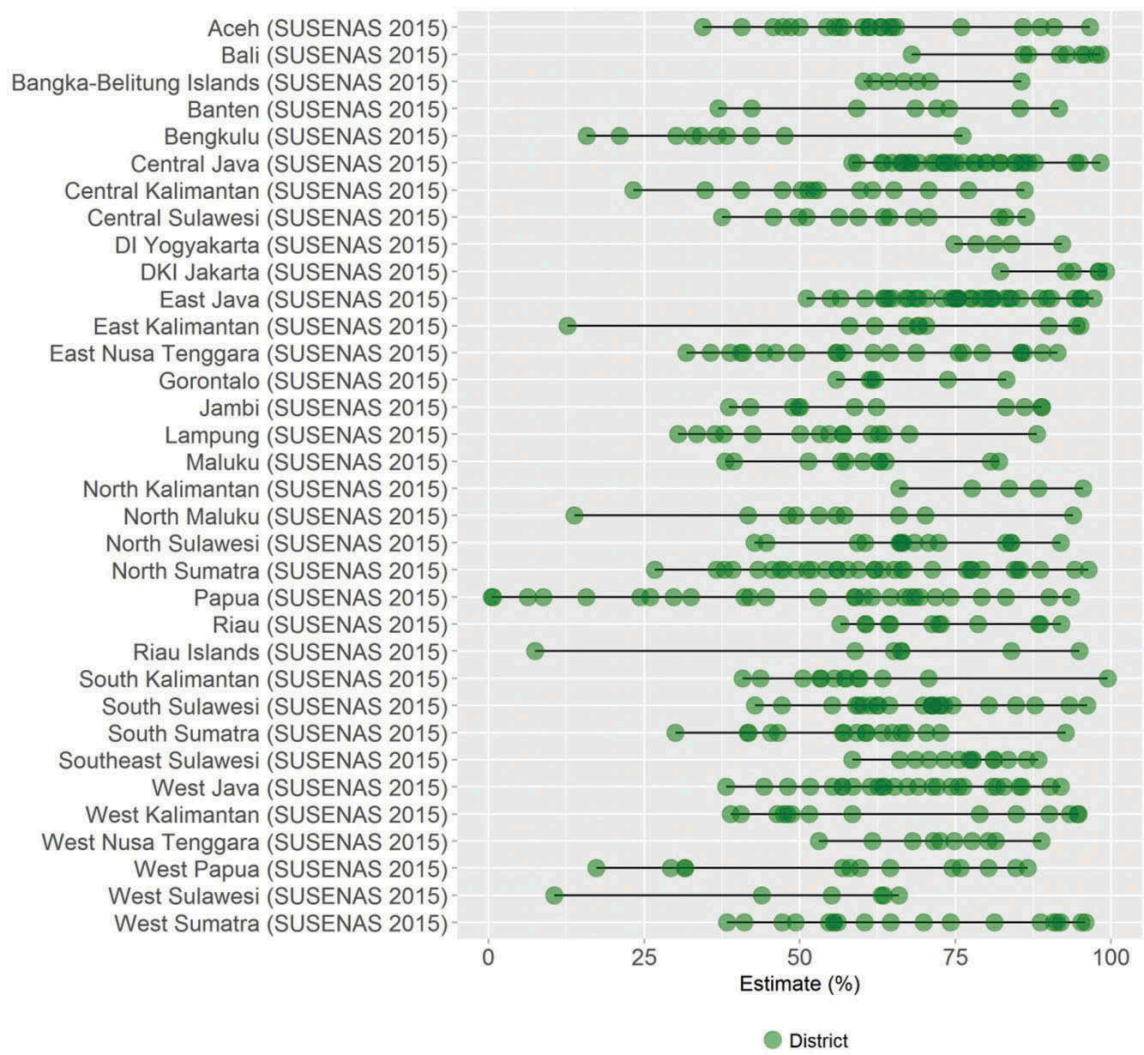
Access to improved drinking water in 34 provinces in Indonesia, by district (SUSENAS 2015).

**Figure 5.**
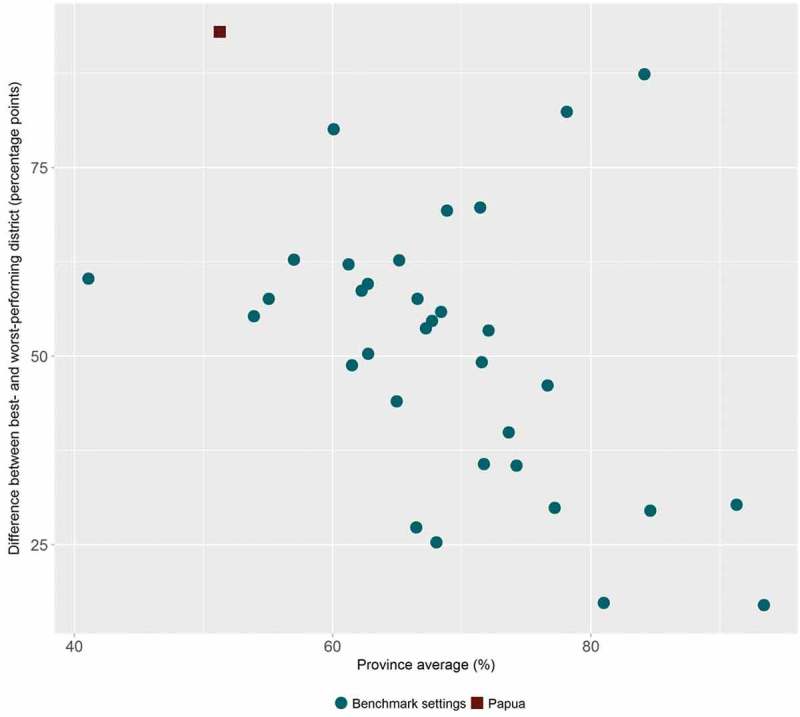
Access to improved drinking water in 34 provinces in Indonesia: province average and absolute within-province inequality (SUSENAS 2015).

**Figure 6.**
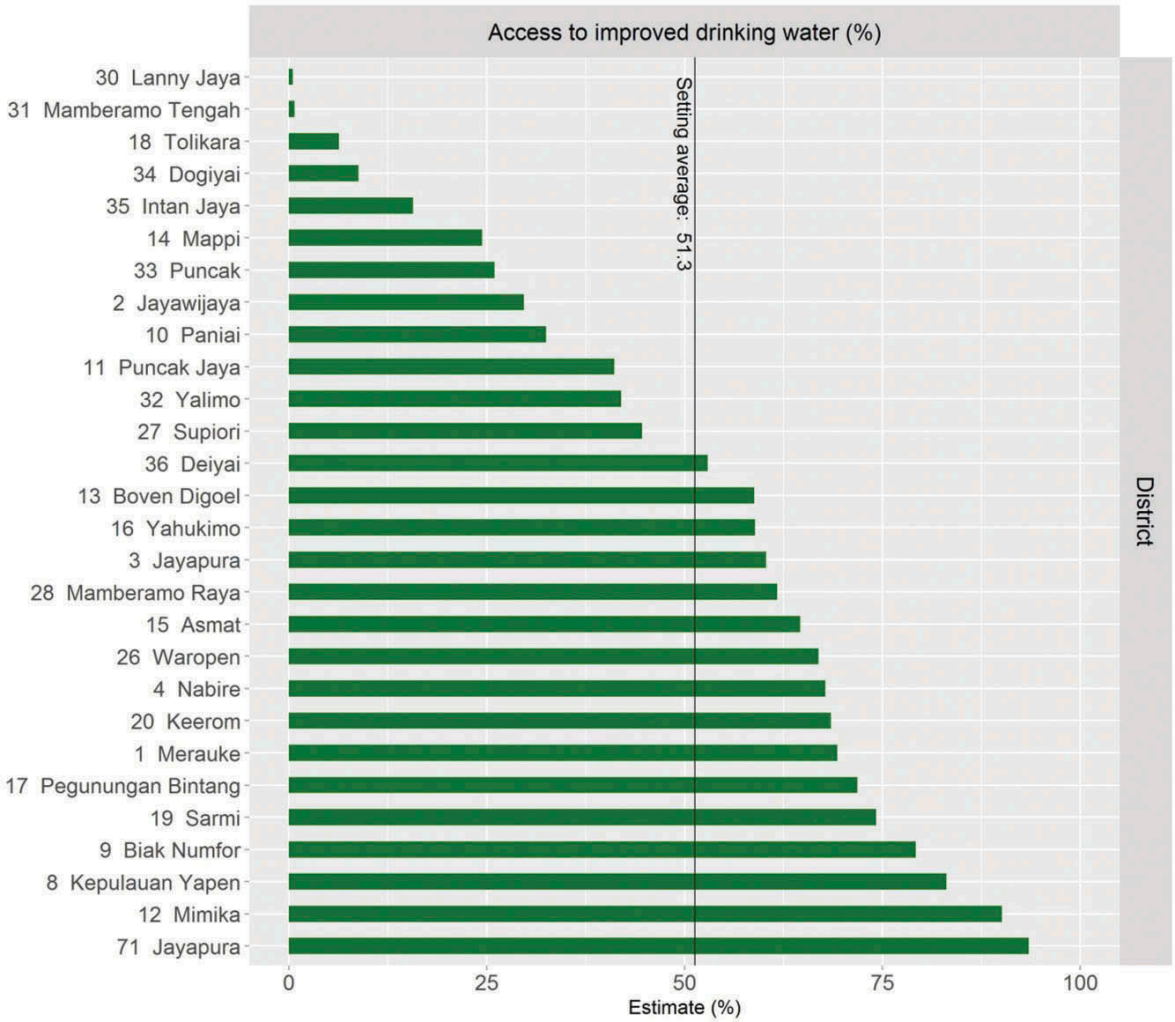
Access to improved drinking water in Papua province in Indonesia, by district (SUSENAS 2015).

HEAT Plus has also been used to assess the state of health inequality at the national level in Indonesia, as part of efforts to build capacity for health inequality monitoring in the country [16]. Data from different national sources, including health facility surveys and household surveys, were used to assess inequalities in selected indicators by relevant dimensions of inequality. For example, data from the 2015 National Socioeconomic Survey (SUSENAS) were used to assess inequalities in access to improved drinking water between districts [17]. Overall, it was found that there were large variations between districts within each province (see Figure 4). Looking simultaneously at the province average and the level of absolute within-province inequality, Papua was identified as one of the provinces with the lowest overall access and the highest absolute inequality (Figure 5). Looking closer at the situation in Papua, it was observed that, overall, about half of the population in Papua had access to improved drinking water, yet in some districts, such as Jayapura, nearly every household had access, while in others, no one had access (Figure 6).

Using HEAT Plus as part of the capacity building process in Indonesia not only facilitated the assessment of the state of health inequality in the country, but also helped to improve the software. For example, users expressed the need to be able to analyse subnational-level data in more detail, which led to the development of the Disaggregated data (detailed bar graphs) tab. Moreover, feedback from users helped to improve the template as well as the warning messages that appear in HEAT Plus if data have not been entered correctly. The results from the capacity building process have been presented in the national report *State of health inequality*: *Indonesia*, published in November 2017, as well as in this special issue [17,18].

## Discussion

While many countries routinely monitor health at the national level, national averages may mask underlying inequalities and population subgroups being left out of health programmes and services. Many countries have disaggregated data available, but lack the tools and capacity to routinely assess health inequalities. HEAT Plus is one of several tools and resources WHO has developed to support global and national health inequality monitoring, such as the *Health Equity Monitor* theme page and data repository, the *Handbook* *on health inequality monitoring: with a special focus on low- and middle-income countries*, the *Health inequality monitoring eLearning module*, the *National health inequality monitoring: a step-by-step manual* and the WHO *Innov8 approach for reviewing national health programmes* [19–24]. The hope is that with HEAT Plus and related tools and resources, countries will be able to incorporate health inequality monitoring as part of their routine health reporting and planning.

Indeed, HEAT Plus shows promise for the adaptation, expansion, and deepening of health inequality monitoring at various administrative levels within countries as the software is highly flexible and adaptable. For instance, once data is prepared following the HEAT Plus template, over time, data may be added for additional periods of time, indicators, dimensions of inequality, sources of data, levels of programming and so on, allowing the analysis  and reporting of equity to be richer, more nuanced and constructive for policymaking and/or programme design. This in turn can help institutionalise consideration of equity in the process, which then must be addressed through programme redesign and refinement [24].

Flexibility and adaptability are perhaps the greatest strengths of this software. Importantly, HEAT Plus allows users to upload and work with their own databases. Health equity assessments can be done at different levels, e.g. at global, national or subnational levels, depending on the data uploaded to HEAT Plus. Assessments can also be done for a variety of indicators, not just in the domain of health (e.g. HEAT Plus can work with health indicators prevalence of a disease, coverage of treatment, services or human resources, as well as non-health indicators like adolescent school attendance, voter turnout, etc.). Similarly, typical dimensions of inequality such as age, sex, education or economic status, but also country- or context-specific dimensions, such as ethnicity, disability or migratory status can be used for the equity assessment. Data used for an assessment in HEAT Plus may come from various types of data sources, such as household surveys, administrative/facility data, census, or civil registration/vital statistics databases. In fact, data from different sources may be linked using individual or small-area unique identifiers and analysed simultaneously [25]. Datasets may be saved securely on the user's machine and analyses generated and downloaded based on user convenience. Finally, once the software is downloaded, internet access is not required to run analyses, making this a useful tool for analyses ‘in the field’ where internet access may be scarce.

The software has incorporated features in response to the requests and requirements of users carrying out health inequality monitoring at the national level, particularly in Indonesia [16]. An email address is provided (heat@who.int), where users may provide feedback to improve the software.

HEAT Plus is in the process of incorporating additional features to enhance its capabilities for equity assessments and to make the software more user friendly. For instance, interactive features will be added to the graphs, allowing users to engage with the data and obtain additional information (e.g. through tooltips that appear when hovering over a data point). A feature is also being added that, should shape files be available, will engage a map function, showing estimates using chloropleth maps. Finally, to ensure wide use and application, HEAT Plus is envisioned to be translated into other languages, with an initial emphasis on languages of the United Nations.

## Conclusion

HEAT Plus, the Upload Database Edition of the Health Equity Assessment Toolkit is a software application that allows users to undertake health equity assessments using their own data. The ability of HEAT Plus to work with uploaded data makes it a suitable tool for both global and national inequality assessments. In particular, the software has direct application for use at the subnational level, enabling customised equity assessment that may be of particular use and relevance for local use and planning. To conclude, HEAT Plus represents a concrete and tangible contribution to the realisation of the SDG vision of leaving no one behind.

## Paper context

In order to attain the Sustainable Development Goals, the World Health Organisation has developed an Upload Database Edition of the Health Equity Assessment Toolkit, a software application that enables the assessment of within-country inequalities in health or beyond. HEAT Plus is open-source and freely available for download from the WHO website. The software can be used to assess inequalities using data from a variety of data sources, for any indicator (health or beyond) and dimension of inequality, following a simple set of preparatory steps. This tool has value for both global and national inequality assessments.
